# The p53 co-activator Zac1 neither induces cell cycle arrest nor apoptosis in chicken Lim1 horizontal progenitor cells

**DOI:** 10.1038/cddiscovery.2015.23

**Published:** 2015-09-07

**Authors:** S Shirazi Fard, MKE Blixt, F Hallböök

**Affiliations:** 1 Department of Neuroscience, Biomedical Center (BMC), Uppsala University, Uppsala, Sweden

## Abstract

Chicken horizontal progenitor cells are able to enter their final mitosis even in the presence of DNA damage despite having a functional p53-p21 system. This suggests that they are resistant to DNA damage and that the regulation of the final cell cycle of horizontal progenitor cells is independent of the p53-p21 system. The activity of p53 is regulated by positive and negative modulators, including the zinc finger containing transcription factor Zac1 (zinc finger protein that regulates apoptosis and cell cycle arrest). Zac1 interacts with and enhances the activity of p53, thereby inducing cell cycle arrest and apoptosis. In this work, we use a gain-of-function assay in which mouse Zac1 (mZac1) is overexpressed in chicken retinal progenitor cells to study the effect on the final cell cycle of horizontal progenitor cells. The results showed that overexpression of mZac1 induced expression of p21 in a p53-dependent way and arrested the cell cycle as well as triggered apoptosis in chicken non-horizontal retinal progenitor cells. The negative regulation of the cell cycle by mZac1 is consistent with its proposed role as a tumour-suppressor gene. However, the horizontal cells were not affected by mZac1 overexpression. They progressed into S- and late G2/M-phase despite overexpression of mZac1. The inability of mZac1 to arrest the cell cycle in horizontal progenitor cells support the notion that the horizontal cells are less sensitive to events that triggers the p53 system during their terminal and neurogenic cell cycle, compared with other retinal cells. These properties are associated with a cell that has a propensity to become neoplastic and thus with a cell that may develop retinoblastoma.

Neurogenesis of the five neuronal cell types: retinal ganglion cells, photoreceptors (PRs), horizontal cells (HCs), amacrines, bipolars, and the Müller glia cell in the retina,^1^ is coordinated by regulation of proliferation, cell cycle exit and differentiation of multipotent retinal progenitors. Retinal progenitors progress through the cell cycle during the process of interkinetic nuclear migration with mitoses on the apical side of the neuroepithelium. Once the cells undergo their final cell cycles, also denoted as the terminal or neurogenic cell cycle, the cells withdraw from the cell cycle and initiate differentiation, while migrating to their final laminar position. This is true for most of the retinal progenitor cells.^2,3^ However, the final cell cycles of Lim homeobox protein 1 (Lim1) expressing HCs are different and some of the mitoses are performed as delayed non-apical mitoses.^4^ Non-apical HC mitoses have been observed in zebrafish and chicken.^5,6^ In chicken, these terminal mitoses take place on the basal side of the retina during a defined period of time,^4,5^ and in combination with the specific expression of Lim1 in HCs, it is possible to specifically study the final cell cycle of these cells.

The cell cycle is regulated by multiple processes, and the DNA damage response pathway, which arrest the cell cycle upon DNA damage and allows time for repair,^7^ has been shown to regulate the cell cycle also in undamaged normal neural stem and progenitor cells.^8–10^ The DNA damage response pathway is engaged and regulate naturally occurring developmental cell death in the retina.^4,11^ The tumour-suppressor protein and transcription factor p53 constitutes a central component of the DNA damage response pathway and arrest the cell cycle by activation of the cyclin-dependent kinase inhibitor 1: p21^CIP1/waf1^ (p21).^12^ Although most retinal progenitors arrest the cell cycle following DNA damage, the chicken retinal horizontal progenitor cells (HPCs) proceeds into their non-apical terminal mitosis in the presence of Cisplatin-induced DNA damage.^13^ The activity of p53 is regulated by multiple positive and negative modulators.^14^ The transcription factor Zac1/Plagl1/LOT1 (zinc finger protein that regulates apoptosis and cell cycle arrest/pleiomorphic adenoma gene-like 1/lost on transformation 1) was identified in several tumours^15–18^ as a tumour-suppressor gene based on its ability to control cell cycle progression and apoptosis.^19^ Furthermore, Zac1 has been shown to interact with and enhance the activity of p53,^20^ providing an explanation to its ability to induce cell cycle arrest and apoptosis. Zac1 was also identified in a screen for genes involved in neural fate specification.^21^ It is expressed in retinal progenitor cells and newly differentiated retinal neurons in the mouse retina, and a Zac1 loss-of-function mutant develops retinal cell hyperplasia. Zac1 was therefore suggested to be a negative regulator of cell number and retina size, which is consistent with a function as a tumour-suppressor gene.^22^

The work presented here was initiated after an experiment where we overexpressed mouse Zac1 (mZac1) in the chicken embryonic retina. The results indicated that while several chicken retinal progenitors were affected by the overexpression of mZac1, chicken Lim1-expressing HPCs were not. This prompted us to specifically study the effect of Zac1 on the final cell cycle of HPCs. The results showed that overexpression of mZac1 induced expression of p21 in a p53-dependent way, and it arrested the cell cycle as well as triggered apoptosis in chicken non-horizontal retinal progenitor cells leading to fewer cells. This effect on the cell cycle in the retina is consistent with the proposed function of Zac1 as a negative regulator of retinal cell generation and as a tumour-suppressor gene. However, the HPCs entered S- and G2/M-phase, and their number did not decrease despite overexpression of mZac1. The inability of mZac1 to arrest the cell cycle in HPCs support our notion that these cells are less sensitive to events that trigger the p53 system during their terminal and neurogenic cell cycle compared with many other retinal progenitors.

## Results

### Overexpression of mZac1 in the chicken embryonic retina

We used a gain-of-function assay based on a mZac1 cDNA expression vector with a cytomegalovirus early enhancer/chicken *β*-actin (CAG)-promoter and a Myc-tag, in combination with *in ovo* electroporation of the chicken embryonic retina. The mZac1 cDNA corresponds to a transcript encoding a 673 amino-acid Zac1 protein that is the predominant transcript in the mouse retina.^23^ Overexpression of this mZac1 sequence has produced a functional response both in mouse and *Xenopus* retina.^22,23^ A pZGs-GFP reporter vector was used either in combination with the mZac1 expression vector to visualize the electroporated area or alone as an electroporation control.

**Figure 1:**
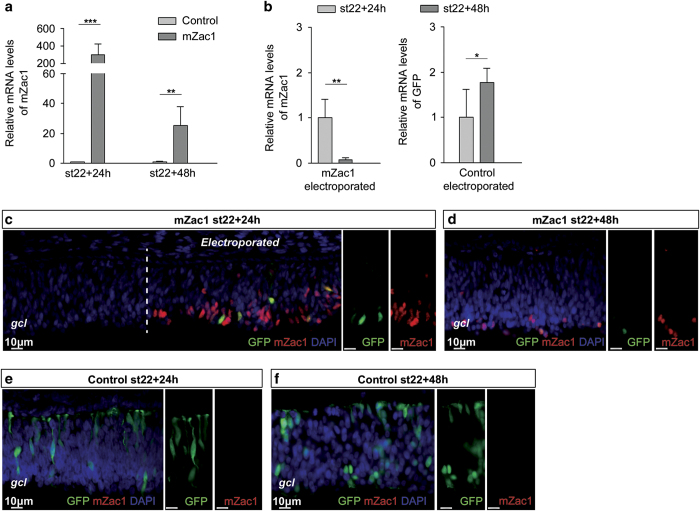
Overexpression of mZac1 in the embryonic chicken retina. Quantitative RT-PCR and immunohistochemistry was used to analyse mRNA and protein expression. (**a**) Relative mZac1 mRNA levels 24 and 48 h after electroporation of mZac1 or control vectors of st22 retinas. (**b**) Relative mRNA levels of mZac1 and GFP 24 and 48 h after electroporation. (**c** and **d**) Fluorescence micrographs of retinas electroporated with mZac1 at st22 and analysed after 24 and 48 h showing mZac1 and GFP IR. Dashed line marks border to mZac1 electroporated region. (**e** and **f**) Fluorescence micrographs of retinas electroporated with the control vector at st22 and analysed after 24 or 48 h. Control: GFP vector, gcl: ganglion cell layer (basal side). Student’s *t*-test, *P<0.05, **P<0.01, ***P<0.001, *N*≥4, mean±S.D.

In Hamburger and Hamilton stage (st) 22 electroporated embryonic retinas, the mZac1 overexpression vector gave a robust 300-fold increase of mZac1 mRNA expression after 24 h compared with control-electroporated retinas. mZac1 mRNA levels were increased after 48 h incubation although at lower levels compared with the 24-h time point (Figures 1a and b). We analysed the GFP mRNA levels in control (pZGs-GFP) electroporated retinas and the levels increased between 24 and 48 h of incubation (Figure 1b). The reduction in mZac1 mRNA was therefore not likely due to transient expression or to the electroporation itself but rather it was likely due to an effect of mZac1. Immunohistochemistry confirmed the presence of mZac1 protein 24 and 48 h after electroporation. mZac1 immunoreactivity (IR) was only present in the electroporated area (Figure 1c). The IR confirmed both the specificity of the mZac1 antibody and the ectopic expression of the mZac1 protein in the electroporated area. Retinas analysed 48 h after electroporation had fewer mZac1 IR-positive (+) cells (1.9±0.4×10^3^/mm^2^, 516 cells counted, *N*=4) compared with after 24 h (6.0±0.4×10^3^/mm^2^, 1316 cells counted, *P*<0.001, *N*=4, Figures 1c and d).

When comparing the expression of co-electroporated GFP and mZac1 plasmids using the mZac1 antibody and the pZGs-GFP expression, we observed that the mZac1 IR localized to regions with GFP fluorescence and that the majority of the GFP+ cells were mZac1+ (>90% co-localization, 676 GFP+ cells counted, Figures 1c and d). In subsequent experiments, the GFP signal was used to identify regions with mZac1-expressing cells. In these regions, the mZac1+ cells were mainly found on the vitreal/basal side of the retina (76±12% of cells on the basal 1/3 of the retina, 1791 cells counted, *N*=4; Figures 1c and d) while in control retinas analysed 24 and 48 h after electroporation, the GFP+ cells were evenly distributed along the apico-basal axis (40±3% of cells on the basal 1/3 of the retina, 2305 cells counted, *N*=4; Figures 1e and f).

The results indicated that overexpression of mZac1 reduced the number of cells, when the 24- and 48-h time points were compared and that the mZac1-expressing cells accumulated on the vitreal/basal side of the retina.

### mZac1-induced p21 expression is blocked by the p53 inhibitor cyclic Pifithrin-*α*

**Figure 2:**
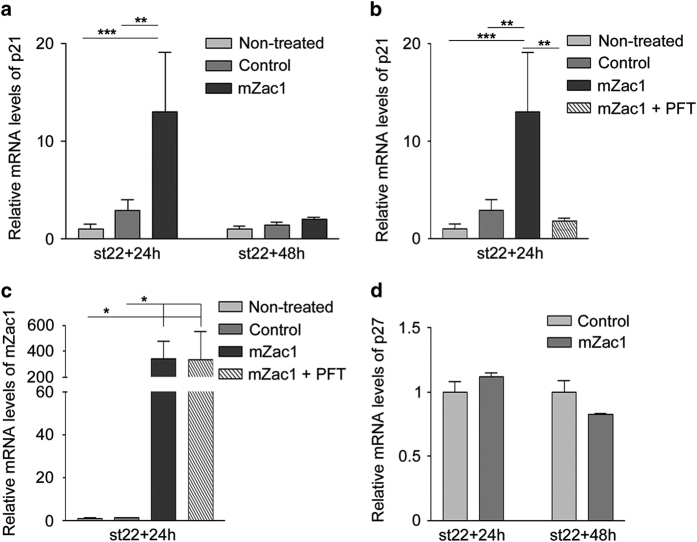
The effect of mZac1 overexpression on the cell cycle regulators p21 and p27. Quantitative RT-PCR analysis of mRNA expression. (**a**) Relative levels of p21 mRNA in non-treated or electroporated (mZac1 or control vector) st22 retinas after 24 and 48 h. (**b**) Relative levels of p21 mRNA in non-treated, electroporated (mZac1 or control vector) and cyclic Pifithrin-*α*-treated st22 retinas after 24 h. (**c**) Relative levels of mZac1 mRNA in non-treated, electroporated (mZac1- or control vector) and cyclic Pifithrin-*α* treated st22 retinas after 24 h. (**d**) Relative levels of p27 mRNA in electroporated st22 retinas after 24 and 48 h. Control: GFP vector, PFT: cyclic Pifithrin-*α*. One-way analysis of variance followed by Tukey’s multiple comparison *post-hoc* test (**a**–**c**) or Student’s *t*-test (**d**), *P<0.05, **P<0.01, ***P<0.001, *N*≥4, mean±S.D.

p53 regulates the cell cycle via transcriptional activation of p21,^24,25^ and Zac1 has been shown to be a co-activator of p53.^26^ We investigated whether overexpression of mZac1 induced p21 expression. p21 mRNA expression was analysed by quantitative reverse transcription PCR (qRT-PCR) at 24 and 48 h after mZac1 overexpression, and p21 mRNA increased 24 h after electroporation compared with both non-treated and control-electroporated retinas (Figure 2a). The results confirmed that mZac1 induces expression of p21 mRNA in the chicken retina. The p21 mRNA expression levels were not significantly increased 48 h after electroporation compared with both non-treated or control-electroporated retinas (Figure 2a).

To investigate whether the mZac1-induced expression of p21 was dependent of p53, we analysed the p21 mRNA expression after blocking p53 with cyclic Pifithrin-*α*.^27^ Retinas were electroporated with mZac1 and cyclic Pifithrin-*α* was injected into the eye, 6 h prior to analysis. Cyclic Pifithrin-*α* blocked the increase in p21 mRNA levels seen after mZac1 electroporation (Figure 2b). To exclude that the effect of cyclic Pifithrin-*α* on p21 expression was due to decreased expression of mZac1, we analysed mZac1 mRNA. The mZac1 mRNA level was similar in retinas treated with cyclic Pifithrin-*α* as without the inhibitor (Figure 2c), indicating that the reduction of p21 was an effect of cyclic Pifithrin-*α*. We analysed the expression of cyclin-dependent kinase inhibitor 1B, p27^Kip1^ (p27),^28^ after mZac1 overexpression. There was no difference in the p27 mRNA levels between mZac1- and control-electroporated retinas (Figure 2d). The results suggested that mZac1 induce p53-dependent expression of p21.

### mZac1 overexpression induced apoptosis

**Figure 3:**
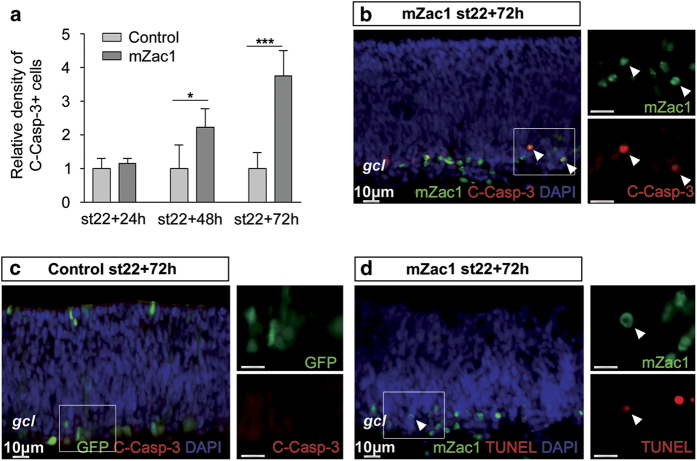
Overexpression of mZac1 induced apoptosis. Apoptosis was analysed by immunohistochemical detection of C-Casp-3+ and TUNEL in mZac1 or control electroporated retinas. C-casp-3+ IR cells were counted. (**a**) Relative density of C-Casp-3+ cells in retinas counted at 24, 48 and 72 h after electroporation. (**b** and **c**) Fluorescence micrographs showing mZac1 and C-Casp-3 IR in mZac1 electroporated st22 retinas, and GFP and C-Casp3 IR in control electroporated st22 retinas, analysed after 72 h. (**d**) mZac1 IR and TUNEL staining in retinas electroporated at st22 and analysed after 72 h. Arrow heads indicate double-positive cells. Control: GFP vector, gcl: ganglion cell layer. Student’s *t*-test, *P<0.05, ***P<0.001, *N*≥4, mean±S.D.

Our data suggest that mZac1 promotes p53 activity, which activates p21 expression, thereby regulating the cell cycle. Zac1 has also been shown to trigger apoptosis.^19^ Our data show a reduction of mZac1+ cells 48 h postelectroporation (Figures 1c and d). Cleaved caspase-3 (C-Casp-3) and TdT-mediated dUTP nick-end labelling (TUNEL) assay was used to analyse apoptosis in the Zac1-electroporated retinas. We counted the number of C-Casp-3 IR cells in the electroporated areas and the number of C-Casp-3 IR cells increased after both 48 h (9.9±2.4×10/mm^2^, 48 cells counted, *N*=4) compared with control-electroporated retinas (4.4±3.1×10/mm^2^, 21 cells counted, *N*=5) and 72 h (18.6±3.7×10/mm^2^, 123 cells counted, *N*=4) compared with control-electroporated retinas (5.0±2.4×10/mm^2^, 22 cells counted, *N*=4; Figures 3a–c), indicating that mZac1 expression induced apoptosis.

Both mZac1, C-Casp-3 and mZac1, TUNEL double-positive cells were seen (Figures 3b and d, arrow heads). Taken together, our data suggest that Zac1-induced p53 activity triggers apoptosis in the electroporated cells.

### Reduction of Visinin+ but not Lim1+ cells by mZac1 overexpression

Lim1+ HCs progress through their final cell cycle and enter the neurogenic mitosis even in the presence of DNA damage,^13^ despite having a functional DNA damage response pathway.^29^ Moreover, normal Lim1+ HCs exhibit heterogeneity during their final cell cycle, producing aneuploid HCs after the S-phase. This behaviour deviates from the behaviour of other retinal cells^4^ and is independent of the DNA damage response with p53. The results showing that Zac1 engages the p53-p21 system prompted us to further investigate the specific effect of Zac1 on the HCs. The majority of Lim+1 HCs in the central retina is generated during the embryonic stages 22–29.^5^ The generation of PRs is also initiated during this period and are derived from the same multipotent retinal progenitor.^30,31^ In the chicken retina, newly formed PRs can specifically be detected using Visinin,^32^ and we counted and compared the Lim1+ cells with the Visinin+ cells in the electroporated regions.

**Figure 4:**
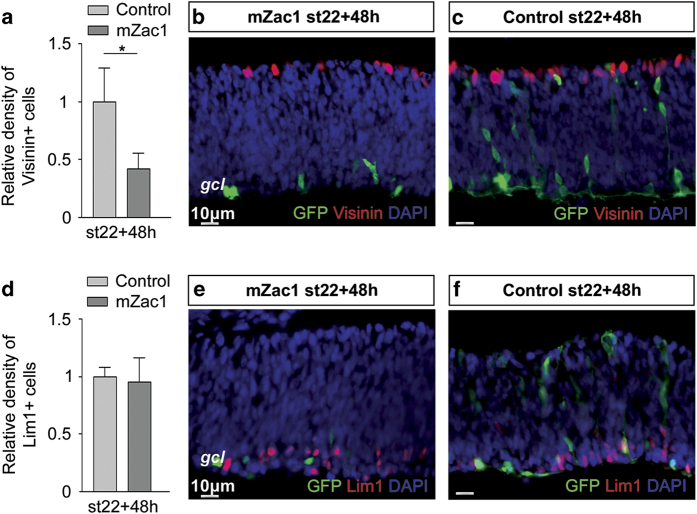
The effect of mZac1 overexpression on the PRs and the Lim1+ HCs. PRs (Visinin) and HCs (Lim1) were analysed using immunohistochemistry in mZac1 and control electroporated st22 retinas. (**a**) Relative density of Visinin+ cells 48 h after electroporation. (**b** and **c**) Fluorescence micrographs showing Visinin+ and GFP+ cells in mZac1 and control electroporated retinas after 48 h. (**d**) Relative density of Lim1+ cells in electroporated retinas after 48 h. (**e** and **f**) Fluorescence micrographs showing Lim1+ and GFP+ cells in mZac1 and control electroporated retinas after 48 h. Control: GFP vector, gcl: ganglion cell layer, Student’s *t*-test, *P<0.05, *N*≥4, mean±S.D.

The number of Visinin+ PRs was lower in the mZac1-expressing regions (7.0±2.2×10^2^/mm^2^, 269 cells counted, *N*=4), compared with the control retinas (16.6±4.8×10^2^/mm^2^, 898 cells counted, *N*=4; Figures 4a–c), whereas there was no difference in the Lim1+ HCs (2.1±0.5×10^3^/mm^2^, 1114 cells counted, *N*=4), compared with the control retinas (2.2±0.2×10^3^/mm^2^, 1033 cells counted, *N*=4; Figures 4d–f). The mZac1 and Lim1 antibodies are both monoclonal, and we used a polyclonal antibody against the Myc-tag to specifically identify and analyse the transfected cells. The lack of effect of mZac1 on transfected Lim1+ HCs was confirmed by counting Myc-tag, Lim1 double-positive cells (5.6±1.1×10^4^/mm^2^, 612 cells counted, *N*=4) compared with control (6.4±2.1×10^4^/mm^2^, 1421 cells counted, *N*=4; non-significant difference *P*=0.55). The results indicate that mZac1 affect the generation of Visinin+ PRs but not Lim1+ HCs.

### mZac1 arrested the cell cycle in retinal progenitors

**Figure 5:**
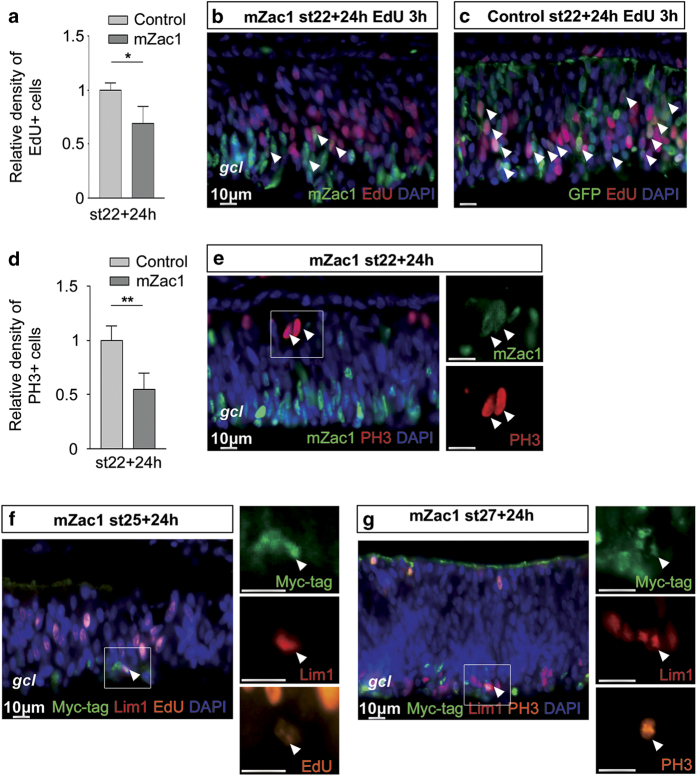
The effect of mZac1 overexpression on cell cycle progression of retinal progenitor cells. The cell cycle progression was analysed using EdU incorporation and PH3 IR in mZac1 and control electroporated retinas. (**a**) Relative density of EdU+ cells in electroporated st22 retinas after 24 h. (**b** and **c**) Fluorescence micrographs showing EdU+ cells in retinas electroporated at st22 with mZac1 or the control vector. (**d**) Relative density of PH3+ cells in electroporated st22 retinas after 24 h. (**e**) Fluorescence micrographs showing PH3+ cells in retinas electroporated at st22 with the mZac1 vector. (**f**) Fluorescence micrographs showing Myc-tag (mZac1), Lim1, EdU IR or in retinas electroporated at st25 with the mZac1 overexpression vector. (**g**) Fluorescence micrographs showing Myc-tag (mZac1), Lim1, PH3 IR in retinas electroporated at st27 with the mZac1 overexpression vector. Arrow heads denote double- or triple-positive cells. Control: GFP vector, gcl: ganglion cell layer. Student’s *t*-test, *P<0.05, **P<0.01, *N*≥4, mean±S.D.

Stage 22 retinas were electroporated with the mZac1 vector and analysed after 24 h. The retinal cells were labelled with 5-ethynyl-2-deoxyuridine (EdU) 3 h prior to analysis. There was a significant decrease in the number of EdU+ cells in the regions with mZac1 overexpression (6.1±1.4×10^3^/mm^2^, 864 cells counted, *N*=4), compared with control (8.8±0.6×10^3^/mm^2^, 1513 cells counted, *N*=4; Figures 5a–c), indicating that the cell cycle in these cells was arrested before entering S-phase. A majority of the mZac1+ cells (67±8%, 1275 cells counted, *N*=4) were localized to the basal side of the retina and were EdU negative (Figure 4b). However, some mZac1, EdU double-positive cells were present (8±3% of mZac1+ cells, 1164 cells counted, *N*=4; Figure 5b, arrowheads), indicating that not all retinal cells arrest after mZac1 overexpression. In the control-electroporated retinas, 34±6% of all GFP+ cells were GFP, EdU double-positive (1232 cells counted, *N*=4; Figure 5c).

We analysed the number of cells entering late G2/M-phase by counting cells that were positive for Phospho-Histone 3 (PH3) 24 h after mZac1 electroporation. A clear reduction of the number of mitoses (6.0±1.7×10^2^/mm^2^, 137 cells counted, *N*=4) was observed compared with control retinas (11.1±8.1×10^2^/mm^2^, 283 cells counted, *N*=4; Figure 5d). However, mZac1, PH3 double-positive cells were seen (Figure 5e, arrow heads), indicating that even though cells expressing mZac1 were arrested, some cells (1.0±0.2% of mZac1+ cells, 1977 cells counted, *N*=4) were able to enter the G2/M-phase while expressing mZac1. Taken together, these data suggest that a majority of the retinal cells that overexpress mZac1 arrest their cell cycle. However, we do observe cells that enter S- and G2/M-phase independently of mZac1 overexpression.

### Lim1+ HCs entered S- and M-phase while overexpressing mZac1

We investigated whether Lim1+ HCs that expressed mZac1 were able to enter S- or late G2/M-phase. We electroporated st25 retinas with the mZac1 vector and analysed after 24 h, during the peak of S-phase performed by Lim1+ HPCs.^4,13^ Cells in S-phase were visualized by EdU incorporation, distributed 6 h prior to analysis. To study the late G2/M-phase, we electroporated st27 retinas with the mZac1 vector and analysed them for PH3 IR after 24 h, corresponding to st29 and the peak in the number of basal mitoses.^4^ The Myc-tag antibody was used to visualize mZac1. Analysis of sections from st27 retinas showed cells that were triple positive for Myc-tag (mZac1), Lim1 and EdU (Figure 5f). The sections from st29 retinas had cells that were triple positive for Myc-tag (mZac1), Lim1 and PH3 (Figure 5g). The results indicated that Lim1+ HCs enter S- and G2/M-phase in the presence of mZac1 overexpression.

### Expression of chicken Zac1 (chZac1) in the developing and injured retina

**Figure 6:**
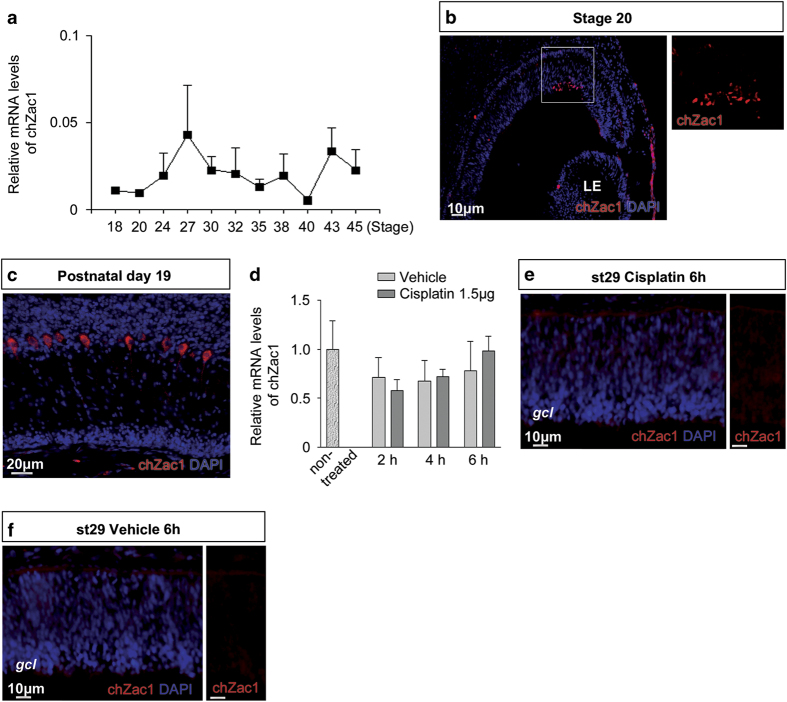
Low expression of chZac1 in the developing and injured retina. Expression of endogenous chZac1 was analysed using qRT-PCR and immunohistochemistry in normally developing and Cisplatin-injured retinas. (**a**) Relative chZac1 mRNA levels in developing retinas of st18–45 chickens. (**b** and **c**) Fluorescence micrographs of chZac1+ cells in st20 embryonic retina and in postnatal day 19 chicken cerebellum. (**d**) Relative chZac1 mRNA levels in non-treated and Cisplatin-treated st29 retinas after 2, 4 and 6 h. (**e** and **f**) Fluorescence micrographs showing absence of chZac1 IR in retinas treated at st29 with Cisplatin or vehicle, and analysed after 6 h. LE: lens, gcl: ganglion cell layer, *N*≥4, mean±S.D.

chZac1 mRNA levels were analysed using qRT-PCR. The levels were low but above background in normal developing retina at all stages (st) 18–45 (embryonic day (E) 3–19; Figure 6a). An antibody that was specific to chZac1 was used for immunohistochemical analysis of embryonic chick retina. The chZac1 IR was weak at all stages investigated ranging from st18 to st45, except at st20 where a cluster of chZac1 IR cells were detected in the dorsal retina (Figure 6b). We confirmed the specificity of the chZac1 antibody by labelling of Purkinje cells in the chicken cerebellum (Figure 6c), where it has been reported to be expressed.^33,34^

Royo *et al*.^35^ exposed cell lines to a DNA damaging reagent for 6 h that resulted in increased Zac1 protein levels. We injected Cisplatin, a chemical reagent that induces DNA damage^13^ and analysed chZac1 mRNA and IR. Cisplatin was injected intraocularly at st29 and chZac1 mRNA levels were analysed after 2, 4 or 6 h. There was no difference in chZac1 mRNA levels between the treated and control retinas (Figure 6d). Furthermore, we did not detect any increased chZac1 IR after Cisplatin treatment compared with control (Figures 6e and f). Cisplatin-induced DNA damage did not trigger chZac1 expression in the chicken retina.

## Discussion

We have studied the effect of Zac1 overexpression in the developing chicken retina. We were particularly interested in the effects on the Lim1-expressing HCs because they exhibit an atypical regulation of their final cell cycle that seems not to be regulated by p53. Our previous results show that Lim1+ HPCs are heterogenic with regards to when and during what cell cycle phase they leave the final cell cycle. There are Lim1+ HPCs that undergo S-phase that is not followed by any mitosis and such cells become aneuploid. Cell death is the most common fate for aneuploid cells,^36,37^ but these HPCs do neither activate any DNA damage response nor do they undergo apoptosis.^4,29^ We have also shown that the HPCs enter mitosis in the presence of DNA damage despite having a functional p53-p21 system.^13^ Here we demonstrate that Zac1 increases expression of p21 in a p53-dependent manner (Figures 2a–c), thereby functioning as a negative regulator of retinal progenitor cells. However, Lim1+ HPCs proceed through their final cell cycle even when they express high levels of Zac1 (Figures 5f and g). The data give support to our hypothesis that these HPCs are less sensitive to events that trigger the p53 system during their neurogenic cell cycle, compared with many other retinal progenitors.

A reduction in the number of cells entering S- and G2/M-phase was observed in mZac1-overexpressing retinas (Figures 5a and d), confirming that Zac1 promotes cell cycle arrest or exit. Furthermore, overexpression of mZac1 resulted in increased mRNA levels of the cyclin-dependent kinase inhibitor p21 in a p53-dependent manner (Figures 2a–c). This provides insights into the possible molecular mechanism involved in Zac1-mediated control of the cell cycle. However, we cannot determine at which stage of the cell cycle the arrest occurs as p21 blocks both the G1/S- and the G2/M-transitions.^38^

Not all of the progenitor cells arrested their cell cycle after overexpression of mZac1 (Figures 4a and d). This could be due to a difference in Zac1 protein expression level after electroporation or that Zac1 might have different functions in different retinal progenitor cells. Zac1 has been suggested to promote proliferation and function as a key regulator of fate decisions in the developing *Xenopus* retina^23^ and in the cerebellum.^34^ This suggests that Zac1 might regulate the cell cycle in a cell type-specific manner. HCs and PRs are among the first retinal cells to be generated during development and they are derived from the same multipotent progenitor during an overlapping period of time.^39^ A comparison was therefore done of the effects of Zac1 expression in HPCs and PRs. The number of PRs was reduced as a result of Zac1 overexpression while, during the same period, the HCs were not affected by Zac1, indicating that the HCs are able to withstand the effect of Zac1 overexpression (Figure 4).

The ability of Zac1 to function as a co-activator of p53 is dependent on association with a functional p53 protein.^20^ We have previously demonstrated that the HCs may trigger a functional p53 response.^13^ However, there seems to be a limitation in the ability of the HPCs to activate p53 after DNA damage. This discrepancy in p53 regulation might also influence the ability of Zac1 to interact with p53 in the HCs. Overexpression of mZac1 in the chicken retina resulted in increased apoptosis (Figure 3a). This is consistent with the ability of p53 to transcribe pro-apoptotic genes leading to cell death.^40^ Our findings in this work confirm the observations from gain-of-function assays in the mouse retina where Zac1 overexpression promotes cell cycle exit and apoptosis.^22,23^ In addition, our results corroborate previous studies reporting that Zac1 promotes cell cycle exit independent of p27.^19^ Spengler *et al*.^19^ reported that the p21 protein levels were unchanged 72 h after Zac1 transfection of a human ostesarcoma cell line. In contrast, we found a robust increase of p21 mRNA 24 h after Zac1 electroporation. The levels were decreased after 48 h and by 72 h we found increased apoptosis, indicating that the p53 system had been activated. We argue that the absent change of p21 after Zac1 expression, reported by Spengler *et al*.,^19^ may be due to the timing of the analysis.

Endogenous Zac1 was expressed at low levels during the entire development of the chicken retina. This is in sharp contrast to Zac1 expression in the developing murine retina, where it is expressed in retinal progenitor cells.^22^ This result suggests that the expression of the Zac1 gene is not evolutionarily conserved. However, it is clear that the chicken cells respond to mZac1. In a previous study,^35^ DNA damage was induced in cell lines that resulted in increased levels of the Zac1 protein. When we induced DNA damage by treatment with Cisplatin, we did not detect any increase of the endogenous chZac1 mRNA or protein (Figures 6d–f) in the retina. This may indicate that the regulation of Zac1 *in vivo* after Cisplatin-induced damage is different to the increase of Zac1 in the mouse fibroblast cell line or that the difference may be a result of posttranscriptional modifications or the level of translation.

In conclusion, the HCs seem to have an atypical regulation of their p53-p21 system not only after DNA damage but also when it comes to the modulators of p53. The p53 co-activator Zac1 functions as a tumour suppressor, and reduced or absent Zac1 expression has been frequently observed in a number of different types of tumours.^15–18^ This is supported by results where cultured retinal explants from Zac1 null allele mice showed an increase in the number of cells in the inner and outer nuclear layer of the retina.^22^ The inability of Zac1 to arrest the cell cycle of HPCs further strengthens the notion that the HCs are less sensitive to signals that regulate cell cycle progression. This is in agreement with a report stating that HCs are able to re-enter the cell cycle, expand clonally and form metastatic retinoblastoma^41^ and may be the ‘cell-of-origin’ for retinoblastoma in that disease model.

## Materials and Methods

### Animals

Fertilized White Leghorn eggs (*Gallus gallus*) were obtained from Ova Production AB (Vittinge, Sweden) and incubated at 37.5 °C in a humidified incubator. Embryos were classified into stages (st) according to Hamburger and Hamilton^42^ or the corresponding embryonic age in days (E).

### DNA constructs

mZac1 expression is driven by the ubiquitous CAG promoter^43^ and the mZac1 is Myc-tagged. The CAG promoter drives gene expression effectively even in differentiated cells.^44^ The GFP expression vector pZGs^45^ was used in combination with the mZac1 expression vector, to visualize the electroporated area or alone as an electroporation control. pZGs also contains the ubiquitous CAG promoter.

### In ovo electroporations

The DNA plasmid (5 *μ*g/*μ*l) was mixed 1 : 1 : 1 with 1×PBS and 0.1 M MgCl_2_. A dye was added to help visualize the injection site. Approximately 0.2 *μ*l DNA plasmid solution was injected into the subretinal space of the embryos and five 50-ms 15-V pulses were applied using an electro square porator ECM 830 (BTX, Harvard Apparatus, Holliston, MA, USA). The eyes were collected after 24, 48 or 72 h and analysed with qRT-PCR and immunohistochemistry.

### Intraocular injections

The thymidine analogue EdU (Click iT EdU Imaging Kit C10337, Life Technologies, Eugene, OR, USA) was used to visualize cells in S-phase.^46,47^ Yolk sac injections were performed with 50 *μ*g EdU. To specifically inhibit p53,^27^ electroporated eyes were injected with 175 ng cyclic Pifithrin-*α* (3843, Tocris, Bristol, UK) 6 h prior to analysis. Cisplatin (2251, Tocris) induces formation of DNA adducts and activates the DNA damage response pathway.^48^ Stage 29 eyes were injected with 1.5 *μ*g Cisplatin 2, 4, or 6 h prior to analysis. The eyes were analysed with qRT-PCR and immunohistochemistry.

### Quantitative reverse transcription PCR

Retinas from the experimental groups or from embryonic stages: st18 (E2½), st20 (E3), st24 (E4), st27 (E5), st30 (E6½), st32 (E7½), st35 (E8), st38 (E12), st40 (E14), st43 (E17), and st45 (E19), were stripped of pigmented epithelium and collected for the qRT-PCR. For ≥st30, the central part of the retina was collected to avoid bias imposed by the centro-peripheral aspects of retinal development. For all treatments/stages, a minimum of four animals were analysed. The mRNA was extracted with Trizol reagent (15596018, Life Technologies). The mRNA batches were treated with DNase (M6101, Promega, Madison, WI, USA) for 30 min at 37 °C before 1 *μ*g of mRNA from each batch was used to prepare cDNA with the high-capacity RNA-to-cDNA Kit (4387406, Life Technologies). The qRT-PCR was run using the IQ SyBr Green Supermix (1708882, Bio-Rad Laboratories AB, Hercules, CA, USA). The initial mRNA levels were normalized to *β*-actin and the TATA box binding protein (TBP). Control reactions containing primers but no cDNA were analysed in parallel. The primers were designed with either Primer Express v2.0 (Applied biosystem, Darmstadt, Germany) or Primer3 Input version 0.4.0 (http://bioinfo.ut.ee/primer3–0.4.0/). Primers used were: chZac1 Fwd: 5′-AAGTGTTCACAGCACGGATG-3′, chZac1 Rev: 5′-TGAAGGCCATCTTGTTTGG-3′; mZac1 Fwd: 5′-AAGTCTCACGCGGAAGAAAA-3′, mZac1 Rev: 5′-CTCTGGGCACAGAACTGACA-3′; p21 Fwd: 5′-CAATGCCGAGTCTGTAGTTCCC-3′, p21 Rev: 5′-TTCCAGTCCTCCTCAGTCCCTT-3′; p27 Fwd: 5′-CCGTCAGAGCCTGGATGTAAA-3′, p27 Rev: 5′-CATCAGTCTTTCGGCCTACACA-3′; *β*-actin Fwd: 5′-AGGTCATCACCATTGGCAATG-3′, *β*-actin Rev: 5′-CCCAAGAAAGATGGCTGGAA-3′; and TBP Fwd: 5′-TAGCCCGATGATGCCGTAT-3′, TBP Rev: 5′-GTTCCCTGTGTCGCTTGC-3′. The data were analysed with one-way ANOVA followed by Tukey’s multiple comparison *post-hoc* test or Student’s *t*-test using GraphPad Prism (v3.02, GraphPad Software Inc., La Jolla, CA, USA), and statistical significance was set to *P*<0.05.

### Immunohistochemistry

Tissues were fixed in 4% paraformaldehyde in 1×PBS for 15 min, followed by 10 min wash with 1×PBS and cryoprotection in 30% sucrose for 3 h, and all steps carried out at 4 °C, before being embedded in freezing medium (NEG50, 6502, Cellab, Sollentuna, Sweden). Tissues were cryosectioned and collected on Superfrost Plus glasses (J1800AMNZ, Menzel-Gläser, Braunschweig, Germany) followed by incubation in blocking solution (PBS containing 1% foetal calf serum and 0.1% Triton X-100) for 30 min. Primary antibodies were allowed to react overnight at 4 °C and secondary antibodies for 2 h at room temperature. Primary and secondary antibodies were diluted in blocking solution. The slides were coverslipped with ProLong Gold with DAPI (936935, Life Technologies) to visualize nuclei. The following antibodies were used: mZac1 (1 : 400, mouse, sc166944, Santa Cruz Biotechnology, Dallas, TX, USA), rZac1 (1 : 200, rabbit, sc22811, Santa Cruz Biotechnology), GFP (1 : 4000, rabbit, ab28283, Abcam, Cambridge, UK), the transcription factor Lim1/2 (1 : 20, mouse, 4F2-s, Developmental studies hybridoma bank (DSHB), Iowa City, IA, USA), Visinin (1 : 1000, mouse, 7G4, DSHB), PH3 (1 : 4000, rabbit, 06–570, Millipore, Darmstadt, Germany), PH3 (1 : 400, goat, sc-12927, Santa Cruz Biotechnology), Caspase-3, cleaved (1 : 4000, rabbit, #9661, Cell Signaling Technology, Danvers, MA, USA), and Myc-tag (1 : 1000, rabbit, C3956, Sigma-Aldrich, St. Louis, MO, USA). Secondary antibodies were obtained from Invitrogen. Samples were analysed using a Zeiss Axioplan 2 microscope (Oberkochen, Germany), equipped with an AxioCam C camera (Oberkochen, Germany) and Axiovision software (Oberkochen, Germany). Images were formatted, resized, enhanced and arranged using Axiovision and Adobe Photoshop CS4 (San José, CA, USA).

### TUNEL staining

TUNEL (G3250, Promega) was used to visualize DNA fragmentation, which indicates apoptotic cell death, according to the manufacturer’s protocol.

### Quantification of S-phases, mitoses and C-Casp-3

Cells in S-phase were labelled with EdU, cells in late G2/M-phase were identified using PH3 IR and cells in the early phases of apoptosis were identified using C-casp-3 IR. At least four sections per eye from four different embryos per treatment and antibody combination were used for cell counting (cells/mm^2^). Only the central part of the retina was analysed to avoid bias imposed by the temporal and centro-peripheral aspects of retinal development. The mean number (±S.D.) for each combination of labelling and stage was calculated, and the data were analysed in GraphPad Prism (v3.02, GraphPad Software Inc.). Analysis of variance was carried out with Student’s *t*-test, and statistical significance was set to *P*<0.05.

### Ethics statement

This study was performed in accordance with the recommendations in the ‘Guide for the Care and Use of Laboratory Animals of the Association for research in vision and ophthalmology’.

## Abbreviations

CAG: cytomegalovirus early enhancer/chicken β-actin promoter
C-Casp-3: cleaved caspase-3
ChZac1: chicken Zac1
EdU: 5-ethynyl-2-deoxyuridine
HC: horizontal cell
HPC: horizontal progenitor cell
IR: immunoreactivity
Lim1: Lim homeobox protein 1
mZac1: mouse Zac1
PH3: Phospho-Histone 3
PR: photoreceptor
qRT-PCR: quantitative reverse transcription PCR
st: Hamburger and Hamilton stage
TBP: TATA box binding protein
TUNEL: TdT-mediated dUTP nick-end labelling
Zac1: zinc finger protein that regulates apoptosis and cell cycle arrest.
